# Rationale and design of the randomized, multicenter, open-label, controlled POLBOS 3 trial aimed to compare regular drug-eluting stents versus the dedicated coronary bifurcation sirolimus-eluting BiOSS LIM C stent

**DOI:** 10.1097/MD.0000000000015106

**Published:** 2019-04-05

**Authors:** Robert J. Gil, Tomasz Pawłowski, Jacek Legutko, Maciej Lesiak, Adam Witkowski, Mariusz Gąsior, Adam Kern, Jacek Bil

**Affiliations:** a Department of Invasive Cardiology, Centre of Postgraduate Medical Education, Warsaw; b Institute of Cardiology, Jagiellonian University Medical College, Krakow; c Department of Cardiology, Poznan University of Medical Sciences, Poznań; d Department of interventional Cardiology and Angiology, Institute of Cardiology, Warsaw; e 3rd Department of Cardiology, School of Medicine with the Division of Dentistry in Zabrze, Medical University of Silesia, Katowice, Silesian Centre for Heart Diseases in Zabrze; f Department of Cardiology and Cardiosurgery, Faculty of Medical Sciences, University of Warmia and Mazury, Olsztyn, Poland.

**Keywords:** BiOSS LIM C, coronary bifurcations, non-LM, sirolimus-eluting stent

## Abstract

**Introduction:**

Coronary bifurcations are encountered in about 15% to 20% of percutaneous coronary interventions (PCIs). They are considered technically challenging and associated with worse clinical outcomes than nonbifurcation lesions. The BiOSS LIM C is a dedicated bifurcation balloon expandable stent made of cobalt-chromium alloy (strut thickness 70 μm) releasing sirolimus (1.4 μg/mm^2^) from the surface of a biodegradable coating comprised of a copolymer of lactic and glycolic acids.

**Conclusion:**

The aim of the randomized, multicenter, open-label, controlled POLBOS III trial is to compare BiOSS LIM C with limus second-generation drug-eluting stents (DES) in the treatment of non-left main stem coronary bifurcations (ClinicalTrials.gov NCT03548272).

## 1 Introduction

The BiOSS (Bifurcation Optimization Stent System) Clinical Program has started in 2008. The first BiOSS stent was a bare metal one (stainless steel), but shortly after a paclitaxel-eluting version was introduced into the market—the BiOSS Expert stent (CE Mark 2010), and in 2012, sirolimus-eluting BiOSS LIM stent was developed. The obtained results both in registries and clinical randomized trials POLBOS I and POLBOS II^[1–6]^ as well as in everyday practice were satisfactory,^[7–9]^ but still a way for improvement has been looking for. Relatively large neointimal growth associated with thick struts (120 μm) was a reason to design a cobalt-chromium sirolimus-eluting version—BiOSS LIM C stent. BiOSS LIM C stent preclinical and preliminary clinical utility has been already confirmed.^[10,11]^

The aim of this trial (POLBOS 3) is to compare BiOSS LIM C with the limus second-generation drug-eluting stents (DES) in the treatment of nonleft main (non-LM) coronary bifurcations.

## 2 Material and methods

### 2.1 Device

The BiOSS LIM C is a dedicated bifurcation balloon expandable stent made of cobalt-chromium alloy (strut thickness 70 μm) releasing sirolimus (1.4 μg/mm^2^) from the surface of a biodegradable coating comprised of a copolymer of lactic and glycolic acids (PGLA). The degradation of the polymer lasts approximately 8 weeks. The BiOSS LIM C stent consists of 2 main separate parts with different diameters: wider proximally and distally smaller. The proximal part is always a shorter than the distal one (45% vs 55%, respectively). The ratio of the proximal part to the distal one varies between 1.15 and 1.3, ensuring physiological compatibility and optimal flow conditions. There is a 2.0 to 2.4 mm middle zone with 2 connecting struts after the BiOSS stent implantation. This zone ensures “self–positioning” of a stent after balloon deflation, as well as the opening to side branch. There are 3 lengths (16, 19, and 24 mm) of BiOSS stents available on the market. The nominal foreshortening of the stent is less than 0.5% and the stent strut/vessel area ratio varies between 15% and 18%.^[12]^

### 2.2 Study population and study design

Five hundred eighteen patients with symptomatic stable coronary artery disease or NSTE-ACS qualified for PCI in non-LM coronary bifurcation will be screened, and enrolled if informed consent form is signed and all inclusion criteria and none exclusion criteria are met. Inclusion criteria and exclusion criteria are detailed in Table 1.

**Table 1:**
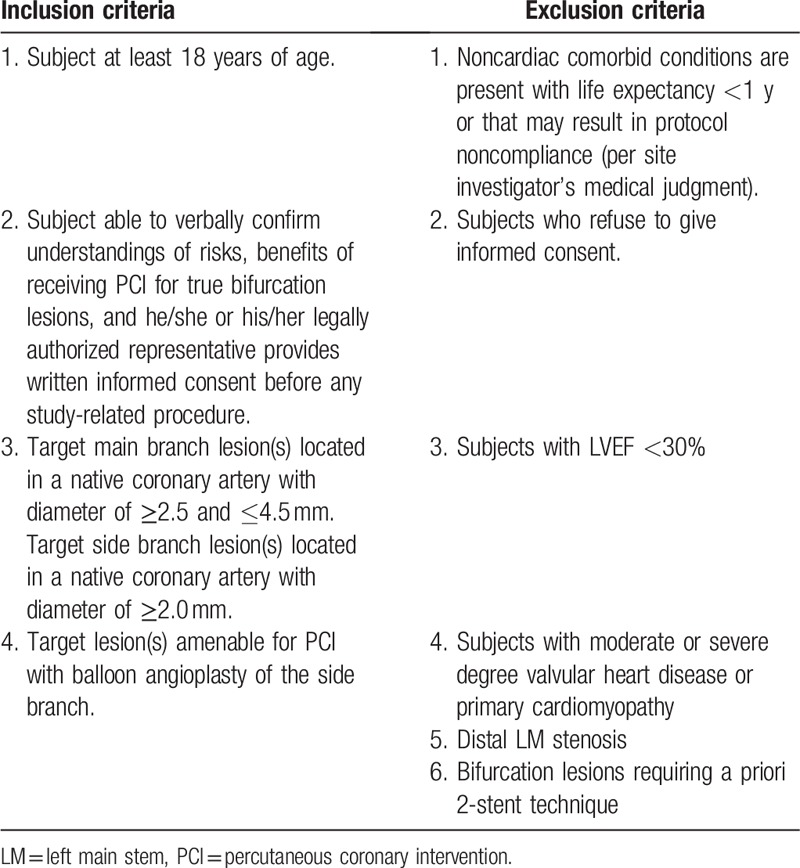
Inclusion and exclusion criteria.

**Figure 1:**
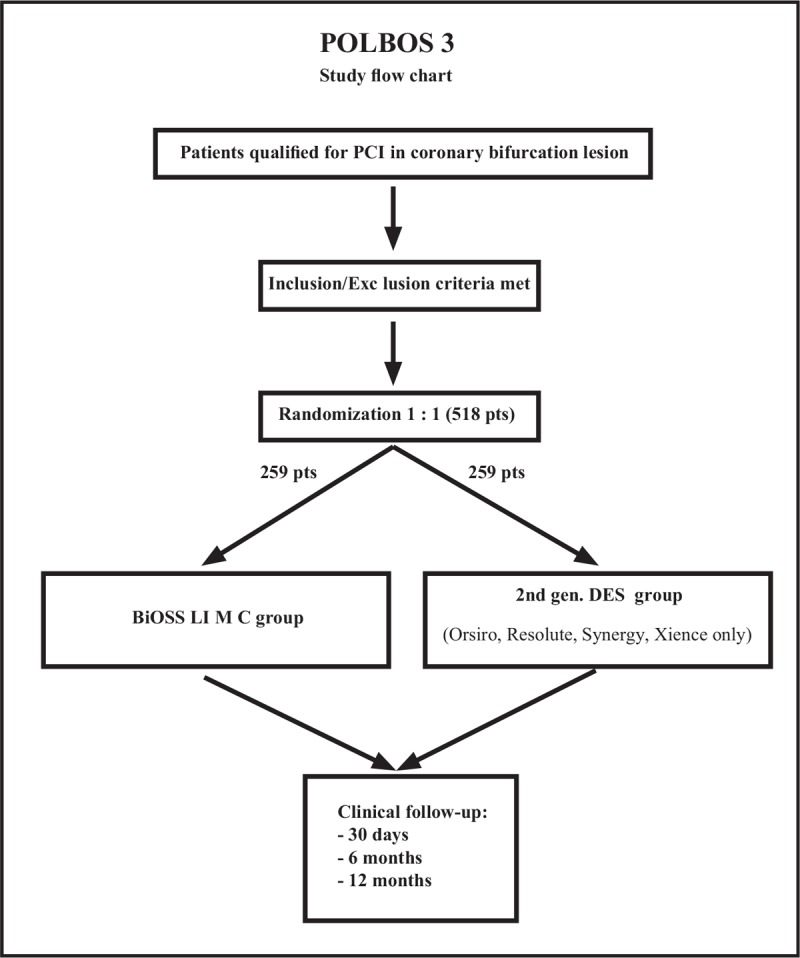
POLBOS 3 flow chart.

Subjects will be 1:1 randomized to a BiOSS LIM C stent versus DES group. The following DES will be allowed: Xience (Abbott Laboratories, Chicago, IL), Resolute Onyx (Medtronic, Dublin, Ireland), Orsiro (Biotronik, Berlin, Germany), and Synergy (Boston Scientific, Marlborough, MA). Patients will be followed, according to the protocol (ClinicalTrials.gov NCT03548272) (Fig. 1). Study protocol is compliant with SPIRIT guidelines.^[13]^ Independent Ethics Committee approved the study protocol (No. 14/2018).

### 2.3 Study methodology

Single stent implantation in the main vessel-main branch across a side branch is the default strategy (provisional T-stenting, PTS) in all patients enrolled. Bifurcation lesions are assessed according to Medina classification using an index of 1 for stenosis greater than 50% and 0 for no stenosis (visual estimation).^[14]^ There is no restriction regarding lesion length in patient selection. If required, additional stent can be implanted (Alex Plus in the BiOSS Lim C Group).^[15]^ A stent in a side branch (Alex Plus in the BiOSS Lim C Group) should be implanted only if there is proximal residual stenosis greater than 70% after balloon dilatation and/or significant flow impairment after main vessel—main branch stenting and/or a flow-limiting dissection.

The implantation protocol for bifurcation is as follows:

(1) wiring of both branches;
(2) main vessel predilatation according to the operator's decision (recommendation BA/MB ratio close to 1:1);
(3) side branch predilatation according to operator's decision, however with small balloon;
(4) stent implantation (inflation for at least 20 seconds);
(5) proximal optimization technique (POT);
(6) side branch postdilatation or side branch stent implantation if necessary (proximal residual stenosis greater than 70% after balloon dilatation and/or significant flow impairment after main vessel—main branch stenting and/or a flow-limiting dissection);
(7) final kissing balloon inflation at operator's discretion (mandatory only if 2-stent technique applied);
(8) Second POT (re-POT)
(9) Additional dilatation of a distal part of a stent (distal optimization technique - DOT) with a NC balloon at operator's discretion.

In patients with ACS, loading dose of ticagrelor (180 mg) or clopidogrel (600 mg) will be given, and if needed also the loading dose of acetylsalicylic acid (ASA) was applied (300 mg). In planned procedures, 72 hours before PCI, each patient will receive ASA (75 mg/24 h) and clopidogrel (75 mg/24 h). All procedures will be performed in a standard way via radial or femoral access using 6 or 7 Fr guiding catheters. After insertion of the arterial sheath, each patient received unfractionated heparin (100 IU/kg). Additional bolus will be given to maintain an activated clotting time > 250 seconds. Dual antiplatelet therapy (ASA 75 mg q.d. quaque die (once a day) and clopidogrel 75 mg q.d. or ticagrelor 90 mg b.i.d. bis in die (twice a day)) will be prescribed for 12 months.

All patients will have TnI, CK, and CK-MB levels examined before the procedure, 6 and 24 hours after. Periprocedural myocardial infarction (type 4a) will be assessed according to the third universal definition (patients with NSTEMI were excluded from this assessment).^[16]^

Quantitative coronary angiography (QCA) analysis before and after the procedure will be performed with the use of the dedicated bifurcation QCA software (CAAS 11.0; Pie Medical Imaging, Maastricht, the Netherlands).

### 2.4 Follow-up

Clinical visits will be performed at 30 days and 12 months, and telephone visit will be performed at 3 and 6 months. At the clinical follow-up visit, clinical status (Canadian Cardiovascular Society Classification), adverse events according to protocol, hospitalization, and medication intake will be evaluated.

### 2.5 Endpoints

The primary endpoint is the rate of major cardiovascular events rate (cardiac death, myocardial infarction, target lesion revascularization) at 12 months. The secondary endpoints are as follows: all-cause death; cardiovascular death—all deaths considered cardiovascular death if not proved otherwise; myocardial infarction—more than 5 times upper limit normal (ULT) increase of troponin, CK-MB, total CK or MRI-detected new areas of late-gadolinium enhancement, associated with symptoms suggesting ischemia, and/or appearance of new-Q waves on surface ECG; target lesion revascularization—any reintervention in previously implanted stent and/or ± 5 mm proximal/distal from stent borers; stent thrombosis—according to Academic Research Consortium definition—definite, probable, possible; early—1 to 30 days after PCI, late—30 days—12 months after PCI, very late—after 12 months after initial PCI, stroke—defined as new persistent neurology deficit, and bleeding—defined according to TIMI group as major and minor.

An independent Clinical Event Committee is built by 3 cardiologists, who are otherwise not involved in the trial. Each clinical event will be adjudicated independently and blindly by 2 members of Committee. In case of disagreement, the third member will be involved and joint agreement should be reached.

### 2.6 Statistics

The mean incidence of MACE rates with currently available stents at 1 year is 10% to 15%. Assuming noninferiority margin (delta) of 8%, a total sample size of 518 patients (with drop-out rate of 5%), 259 patients per group, will be necessary (type I error: 0.05, type II error: 0.2, statistical power 80%). During the analysis, continuous variables in the 2 groups will be compared by unpaired Student *t* test, Mann–Whitney test, or analysis of variance (ANOVA) test, as appropriate. Categorical variables in the 2 groups will be compared with Fisher exact or Chi-square tests, as appropriate. Difference in event-free survival between the 2 groups will be evaluated by applying the Kaplan–Meier curves. Results will be adjusted by Cox-regression multivariate analysis where needed, as appropriate. A probability value of *P* < .05 is considered as statistically significant.

### 2.7 Data reporting

Central data management will be performed by principle investigators at the Centre of Postgraduate Medical Education in Warsaw. Data will be recorded at each site on a case report form, verified primarily by the local investigator. It is the investigators’ responsibility to ensure the accuracy, completeness, and legibility of the data collection. Source documentation should indicate dates and details of routine examinations, treatment interventions, and adverse events.

## 3 Discussion and conclusion

Coronary bifurcations are encountered in about 15% to 20% of percutaneous coronary interventions. They are considered technically challenging and associated with worse clinical outcomes than nonbifurcation lesions. The optimal treatment is still the matter of debate and the use of dedicated bifurcations stents is also a matter of discussion.^[17,18]^ The BiOSS LIM C is a dedicated bifurcation balloon expandable stent made of cobalt-chromium alloy (strut thickness 70 μm) releasing sirolimus (1.4 μg/mm^2^) from the surface of a biodegradable coating comprised of a copolymer of lactic and glycolic acids. The aim of the randomized, multicenter, open-label, controlled POLBOS III trial is to compare BiOSS LIM C with olimus second-generation drug-eluting stents in the treatment of non-LM coronary bifurcations (ClinicalTrials.gov NCT03548272).

## Author contributions

**Conceptualization:** Robert J. Gil, Jacek Bil.

**Formal analysis:** Jacek Bil.

**Methodology:** Robert J. Gil, Tomasz Pawłowski, Jacek Legutko, Maciej Lesiak, Adam Witkowski, Mariusz Gąsior, Adam Kern, Jacek Bil.

**Writing – Original Draft:** Robert J. Gil, Jacek Bil.

**Writing – Review & Editing:** Robert J. Gil, Tomasz Pawłowski, Jacek Legutko, Maciej Lesiak, Adam Witkowski, Mariusz Gąsior, Adam Kern, Jacek Bil.
